# Research on Self-Noise Characteristics of Nine Types of Seismometers Obtained by PDF Representation Using Continuous Seismic Data from the Malingshan Seismic Station, China

**DOI:** 10.3390/s23010110

**Published:** 2022-12-22

**Authors:** Kaiming Wang, Wenyi Li, Lijun Zhao, Daxin Yu, Shaogang Wei

**Affiliations:** 1 The First Monitoring and Application Center, China Earthquake Administration, Tianjin 300180, China; 2 School of Materials Engineering, Changshu Institute of Technology, Changshu 215500, China

**Keywords:** self-noise, seismometer, coherence analysis, power spectral density, probability density function, microseism band

## Abstract

The self-noise level of a seismometer can determine the performance of the seismic instrument and limit the ability to use seismic data to solve geoscience problems. Accurately measuring and simultaneously comparing the self-noise models from different types of seismometers has long been a challenging task due to the constraints of observation conditions. In this paper, the self-noise power spectral density (PSD) values of nine types of seismometers are calculated using four months of continuous seismic waveforms from Malingshan seismic station, China, and nine self-noise models are obtained based on the probability density function (PDF) representation. For the seismometer STS-2.5, the self-noise levels on the horizontal channels (E–W and N–S) are significantly higher than that on the vertical channel (U–D) in the microseism band (0.1 Hz to 1 Hz), which is a computing bias caused by the misalignment between the sensors in the horizontal direction, while the remarkably elevated noise on the horizontal channels at the low frequencies (<0.6 Hz) may originate from the local variation of atmospheric pressure. As for the very broadband seismometers Trillium-Horizon-120 and Trillium-120PA, and the ultra-broadband seismometers Trillium-Horizon-360 and CMG-3T-360, there is a trade-off between the microseism band range and low-frequency range in the PSD curves of the vertical channel. When the level of self-noise in the microseism band is high, the self-noise at low frequencies is relatively low. Although compared with the other very broadband seismometers, the self-noise level of the vertical component of the STS-2.5 is 3 dB to 4 dB lower at frequencies less than 1 Hz, the self-noise level of the STS-2.5 at high frequencies (>2 Hz) is slightly higher than others. From our observations, we conclude that the nine seismometers cannot reach the lowest noise level in all frequency bands within the working range.

## 1. Introduction

Noise is an ever-present signal in digital seismograph records. The recorded noise signal mainly includes natural seismic ambient noise, anthropogenic noise, and instrumental self-noise. Natural seismic ambient noise comes from several sources, such as marine microseisms and changes in atmospheric pressure, temperature, and the back-ground geomagnetic field. Anthropogenic noise is mainly high-frequency noise signals (>1 Hz). The self-noise of a seismograph characterizes the threshold value of the minimum vibration the instrument can detect. Seismographs with lower noise levels can record higher quality data and provide more wave-field information, while those with high noise levels limit the ability to identify and extract more microseismic signals. It is important to evaluate the contribution of the instrumental self-noise to the seismic recording before conducting research on seismic ambient noise fields. For example, when imaging the sub-surface structure of the Earth through seismic ambient noise tomography [1,2,3], the self-noise of the instrument must be lower than the seismic ambient noise of the Earth. Furthermore, the self-noise level is an important indicator in evaluating the performance of the observation system. Self-noise can also serve as a diagnostic measure for seismograph operation.

The self-noise of a digital seismograph is derived from the seismometer (sensor) and the digital datalogger, with the noise of the latter being lower [4]. When the digital datalogger is set to a high gain mode, its self-noise is much lower than that of the seismic sensor in the frequency band of interest [5]. The estimation of the sensor self-noise inevitably contains the datalogger noise; however, its noise is negligible compared with the seismometer. In real earthquake monitoring, the monitoring capability of a seismograph depends mainly on the performance of the sensor, which explains why research on digital seismograph self-noise mainly focuses on seismometers [6,7,8].

Coherence analysis is the main method for calculating seismometer self-noise. Based on coherence analysis techniques, various approaches are employed for extracting self-noise, including the two-sensor method [7,9,10] and three-sensor method [11]. When using the coherence analysis technique, multiple factors could affect the estimation of the instrumental self-noise, such as the calculation method, variability of instrument quality, and the site environment and installation method [4,12]. With the three-channel coherence analysis technique, Ringler and Hutt [4] produced a self-noise model of 11 different seismometers models based on approximately 10 h of continuous waveforms. As the self-noise of a seismometer could be time-dependent, a short-duration recording could lead to random calculation results for the noise power spectral density (PSD). Selecting adequate reliable waveform data is challenging when the recorded waveform contains incoherent gaps or signals with a high signal-to-noise ratio (SNR). For waveforms recorded over a longer period, a more robust noise estimate could be obtained by using the probability density function (PDF) analysis of the PSD [13]. A strict experimental environment and long-duration continuous observation could facilitate more accurate analyses of changes in the self-noise of a seismometer. However, space and time requirements make simultaneous measuring of different types of seismometers difficult. Although a standard method could be used to calculate the incoherent noise of instruments for comparing the self-noise levels of different seismometers obtained from different tests, differences in the experimental environment, such as the station environment and the seismometer installation method, remain non-negligible constraints.

At present, there exist dozens of different types of seismometers within China, which are widely used simultaneously in various seismic monitoring and geophysical studies. Due to a lack of consensus on the working performance of these seismometers, for a long time, the quality of the corresponding seismic observation data has not been adequately evaluated. The differences in seismic observations may lead to potential uncertainties in geophysical interpretations. For the purpose of comparing the self-noise levels of different types of seismometers deployed in China, the China Earthquake Administration conducted a comparison test on their performance at the Malingshan Seismic Station in 2018. In this paper, we calculated the self-noise models of nine types of seismometers among the types in that test. The continuous waveforms of these nine types of seismometers were selected, and the PDF representation method was adopted to calculate their self-noise PSD. As these seismometers were installed in approximately the same experimental environment, and the data were recorded during the same period, we could reasonably compare their self-noise levels. Our results may serve as a reference for procurement standards of seismometers and the evaluation of their performance, as well as provide a better understanding of Earth studies with less uncertainty.

## 2. Materials and Methods

### 2.1. Two- and Three-Sensor Coherence Analysis

As the noise recorded by a seismometer comprises local ambient noise and the self-noise of the seismometer, the key to estimating the self-noise is to remove the ambient noise. Holcomb [9] proposed the two-sensor coherence analysis technique to estimate self-noise. This method assumes that two collocated coaligned seismometers record exactly the same seismic signal, the self-noises of different seismometers are incoherent, and the self-noise is incoherent with the input signal. Based on this assumption, the autocorrelation of one seismometer record consists of the input seismic signal and the self-noise of the seismometer. The cross-correlation of different seismometer records could be attributed mainly to the input seismic signal, i.e., the seismic ambient noise. Therefore, the incoherent seismometer self-noise can remain after removing the cross-correlation between two sensors from the autocorrelation of the sensor. In the frequency domain, calculating the power spectrum $P_{ii}$ and $P_{jj}$ from sensors *i* and *j*, as well as the cross-spectrum $P_{ij}$ between the two sensors, the self-noise $N_{ii}$ of sensor *i* can be expressed as:

(1) $$N_{ii}=\frac{1}{{\left|H_{i}\right|}^{2}}(P_{ii}-\frac{H_{i}^{*}}{H_{j}^{*}}P_{ij})$$

where $H_{i}$ and $H_{j}$ are the transfer function of the two seismometers. Equation (1) shows that the two-sensor method requires the accurate relative transfer function $H_{i}/H_{j}$ of the seismometers. Errors in the transfer function could lead to errors in the estimation of self-noise [7,10]. If the transfer functions of the two seismometers were exactly the same, uncertainty in the transfer function of a seismometer would have a relatively negligible effect on estimating the sensor self-noise. However, in real settings, exactly the same transfer functions of even the same model seismometers cannot be guaranteed.

Following the same assumption as in the two-sensor method, Sleeman et al. [11] proposed a three-channel coherence analysis technique to extract the self-noise of three collocated coaligned seismometers. Because of the third collocated seismometer k, the relative transfer function $H_{i}/H_{j}$ between sensors *i* and *j* can be replaced by the ratio of the cross-PSD between these two seismometers and the third seismometer *k*, i.e., $H_{i}/H_{j}=P_{ik}/P_{jk}$. Therefore, the self-noise of seismometer *i* can be expressed as:

(2) $$N_{ii}=\frac{1}{{\left|H_{i}\right|}^{2}}(P_{ii}-\frac{P_{ik}}{P_{jk}}P_{ij})$$

Equation (2) shows that the three-sensor method is not sensitive to errors in the seismometer transfer function, as the relative transfer function does not need to be measured. Compared with the two-channel coherence analysis technique, the three-sensor method minimizes the errors caused by inaccurate estimation of the seismometer transfer function and can produce a self-noise estimate that is more accurate. The results of Ringler et al. [12] show that the three-sensor method is superior in removing coherent ambient noise and extracts lower instrumental self-noise. We used the three-channel coherence analysis technique in this study to calculate the self-noise of the seismometers.

### 2.2. Seismometer Installation and Data Logging

**Figure 1:**
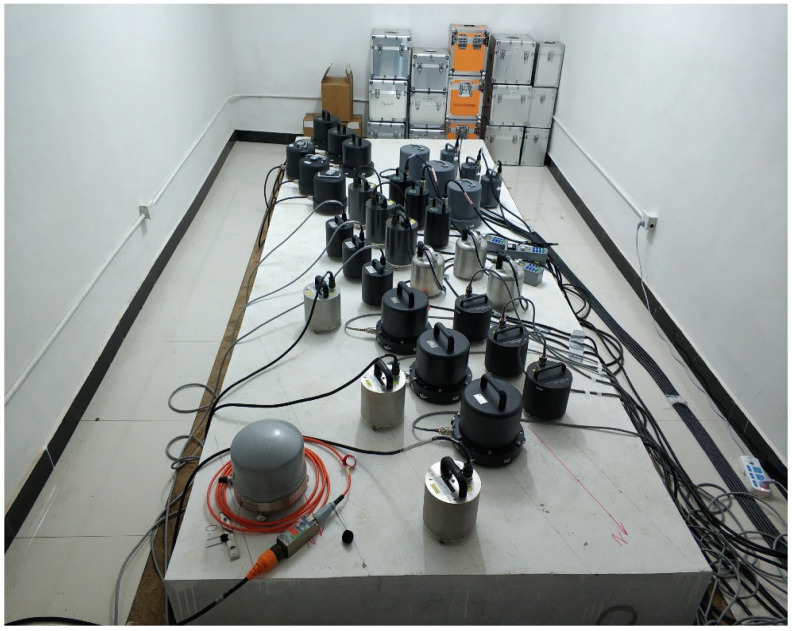
Installation of short-period seismometers and broadband seismometers.

The Malingshan seismic station is located on the west side of Maling Mountain in Tancheng, Shandong Province, China, and its observation rooms are converted from a series of underground tunnels. The tunnels are kept at 16 °C year-round, with an annual temperature difference of no more than 0.3 °C, and also feature low seismic background noise. In 2018, the China Earthquake Administration conducted a test of seismometer self-noise levels at the Malingshan seismic station. The test covered 26 different types of seismic instruments, three of each type, for a total of 78 seismometers. All seismometers were installed in three adjacent underground tunnels at the Malingshan seismic station. There were one or two observation piers in each tunnel, and three sensors of one type were collocated and coaligned on the same pier (Figure 1). Following the installation method of Sleeman and Melichar [13], very broadband and ultra-broadband seismometers were equipped with a neoprene heat shield, which can also be effective in blocking air flow. The seismic data loggers used in this test were the EDAS-24GN from Beijing Geolight Technology Co., Ltd, China, with a sampling rate of 100 samples per second (sps) and a range of ±2.5 V. The ±2.5 V setting of the EDAS-24GN is similar to the high-gain mode of the Quanterra Q330 and Retek 130. The EDAS-24GN has two data acquisition channels, allowing two seismometers to be connected synchronously. By using 42 sets of EDAS-24GN, seismic observations were carried out from 22 November 2018–26 March 2019, and 125 days of continuous data were obtained from 78 seismometers.

In this study, we selected 9 types of seismometers from the 26 types at the Malingshan Seismic Station (Table 1), among which were short-period, broadband, very broadband and ultra-broadband instruments. The other 17 types were not included at the request of the vendors. As settling-down time is needed for seismometers to stabilize after installation to release mechanical stress, we discarded the continuous recording of the first three days after the seismometer started functioning, and ultimately, the data continuously recorded from 25 November 2018–25 March 2019 were used.

### 2.3. PSD Calculation and PDF Representation

According to Equation (2), the three-channel coherence analysis technique requires the calculation of the PSD of the seismometer and the cross-PSD between seismometers of the same type. The PSDs are calculated by using Welch’s averaged periodogram method [14]. Referring to Evans et al. [15] and the China Earthquake Administration [16], the calculation parameters vary in data segment length, overlap rate, and window length (or sub-segment length) for different types of seismometers. Table 1 shows the calculation parameters of the PSDs of the different types of seismometers. For example, for a broadband seismometer, the continuous waveform data of each channel are divided first into segments of data with a length of 1 h and overlap of 50%. Subsequently, according to the Welch [14] method, the PSDs is calculated for each 1 h of data segment. The detailed calculation process is as follows: (1) Divide the 1 h data segment into 14 sub-segments, with a length of 2^17^ samples, which means that the window length is 1310.72 s and the overlap of adjacent sub-segments is 7/8. (2) Remove the mean of the time series of the sub-segment, remove the long-period trend, and apply the normalized Hanning window to calculate the PSD. (3) Average the PSDs of the 14 sub-segment data to obtain the one-sided PSD of the 1 h data segment. Finally, deconvolve with the instrument response of the sensor to obtain the PSD curve relative to acceleration. Since the window length of the Fourier transform is 1310.72 s, and the sampling rate of the waveform data is 100 sps, the frequency band range of the PSD of the broadband seismometer is between 0.0008 and 40 Hz. As is shown in Table 1, for a short-period sensor, the sub-segment or window length is 163.84 s (2^14^ samples), and the PSD frequency band range is between 0.006 and 40 Hz; for an ultra-broadband sensor, the window length is 5242.88 s (2^19^ samples), and the PSD frequency band is 0.0002 and 40 Hz. 

The seismometer self-noise PSD curve of data segments at different times can be obtained by using Equation (2). To study the overall characteristics of the seismometer self-noise level, we referred to the Sleeman and Melichar [13] method and used PDF to characterize the statistical distribution of the seismometer self-noise PSD in the observation period. According to McNamara and Buland [17], the PDF of PSDs can be represented as:

(3) $$P(f_{n},dl)=\frac{N(f_{n},dl)}{N(f_{n},ds)}$$

where d*s* is the entire statistical range of PSD values, d*l* is the *l*-th unit interval in the statistical range of ds, $N(f_{n},ds)$ represents the number of all PSDs in the d*s* at frequency of $f_{n}$, and $N(f_{n},dl)$ is the number of PSDs in the d*l* at the frequency of $f_{n}$. In this study, the number of PSDs within the range −240–−60 dB was counted at intervals of 1 dB, whereby the statistical distribution of the seismometer self-noise PSD during the four months of observation was obtained. In the PDF distribution diagram, the mode represents the PSD value corresponding to the highest probability at different frequency points, which indicates the most common self-noise level of the sensor during the observation period.

The estimated sensor self-noise may have a large bias when the waveform data have incoherent gaps between different data streams or contain signals with high SNR, such as seismic signals [13]. For improved measuring accuracy, we selected the calculated PSD curves with reference to the data selection criteria of Sleeman and Melichar [13], following serveral steps. First, we calculated the PDFs mode, subsequently discarded the PSD curves with a power spectral value of 30 dB greater than the mode at a certain frequency and, finally, re-computed the PDF distribution of PSD.

## 3. Results

This section presents the PDF distributions of self-noise PSDs for nine types of seismometers, which are obtained from continuous seismic observations from 25 November 2018–25 March 2019. For the Streckeisen STS-2.5, the PDF distributions of PSDs are given for the three channels (U–D, E–W and N–S); for the other eight sensors, the PDF distributions are given for the vertical channels (U–D).

### 3.1. Streckeisen Seismometers

**Figure 2:**
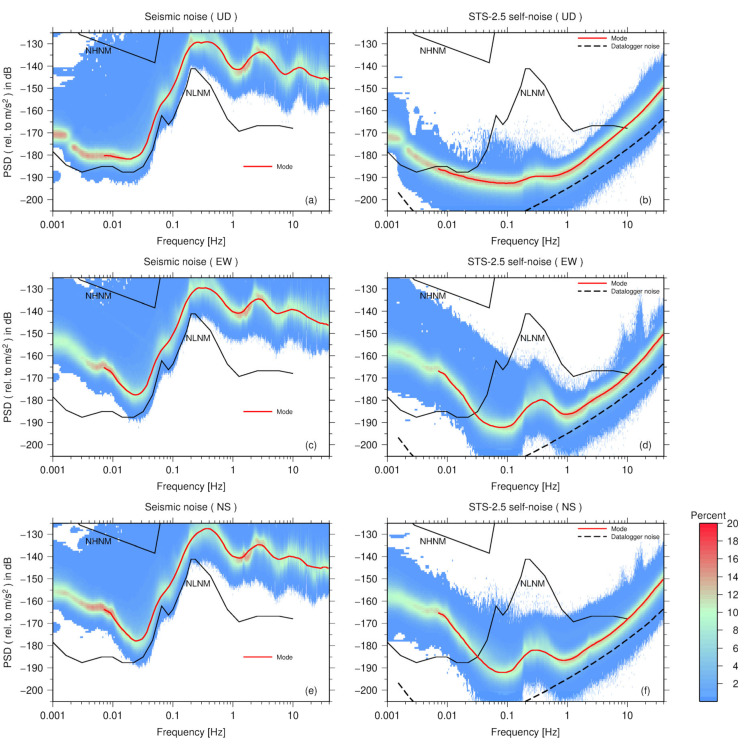
PDF distributions of seismic ambient noise PSD and STS-2.5 self-noise PSD. (**a**), (**c**), and (**e**) are the PDF distributions of background noise of U–D, E–W, and N–S channel estimated by STS-2.5; (**b**), (**d**), and (**f**) are the PDF distributions of STS-2.5 self-noise of U–D, E–W, and N–S channels. The red curve is the mode curve of the PSD after 1/8 octave smoothing. The black dashed curve represents the EDAS-24GN self-noise PSD after smoothing. The black solid curves represent the New High-Noise Model (NHNM) and New Low-Noise Model (NLNM).

The STS-2.5 is a broadband seismometer with a flat velocity response between 120 s and 50 Hz from Streckeisen GmbH, Switzerland (Streckeisen). Figure 2a,c,e show the PDF distributions of the seismic ambient noise PSD for the three channels (U-D, E-W and N-S) calculated by one STS-2.5. Figure 2b,d,f is the PDFs of the STS-2.5 self-noise PSD of the three channels, respectively. The red curves in Figure 2 are the mode curves of the PSD distribution smoothed by 1/8 octave. The black solid curves represent the New High-Noise Model (NHNM) and New Low-Noise Model (NLNM) [18], which are commonly used to represent the high and low noise boundaries of ground motion acceleration. The percentage scale of probability density ranges from 0 to 20 percent, as shown in the color bar at the right side of Figure 2. Actually, we obtained the self-noise models for each of the three STS-2.5s by the three-sensor method, and they are in good agreement (in Appendix A). Variable quality between the sensors can be ignored.

The self-noise of the digitizer is required to be lower than of the seismometer before evaluating the sensor noise. By connecting a 50 ohm resistor to the input of the EDAS-24GN, we also calculated the self-noise PSD of the EDAS-24GN. In order to compare with the seismometer, the self-noise PSD (V^2^/Hz) of EDAS-24GN is converted to the acceleration PSD ((m/s^2^)^2^/Hz) by the instrument response and the voltage sensitivity (Table 1). As is shown in Figure 2b, the EDAS-24GN self-noise level (black curve) is well below the mode of the STS-2.5 noise and, therefore, it is a suitable choice to use the digitizer EDAS-24GN to extract the self-noise of the seismometer in this test.

### 3.2. Güralp Seismometers

**Figure 3:**
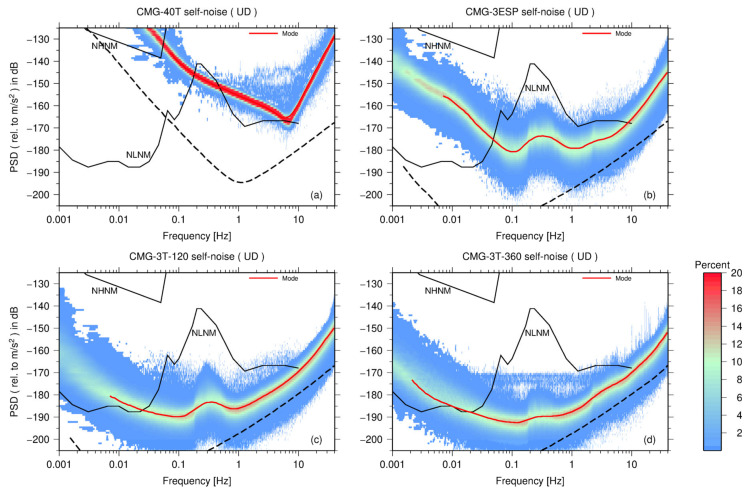
PDF distributions of self-noise PSD (on U–D channel) of four types of seismometers from Güralp. (**a**) CMG-40T, (**b**) CMG-3ESP, (**c**) CMT-3T-120, and (**d**) CMT-3T-360. The red curve is the mode curve of the PSD after 1/8 octave smoothing. The black dashed curve represents the EDAS-24GN self-noise.

There are four seismometers from Güralp Systems Ltd., United Kingdom (Güralp). CMG-40T in this experiment is a short-period seismometer with a frequency band from 2 s to 100 Hz. There are also two types of broadband sensors: the CMG-3ESP has a flat velocity response from 60 s to 50 Hz; the CMG-3T-120 has a wider band range with a low-end cutoff frequency of 120 s. The CMG-3T-360 is an ultra-broadband instrument with a flat velocity response between 360 s and 50 Hz. The self-noise PDF representations on the U–D channel of this seismometer are shown In Figure 3. Because of the space in this paper, the self-noise models for the horizontal channels (E–W and N–S) are given in Appendix B.

### 3.3. Nanometrics Inc. Seismometers

**Figure 4:**
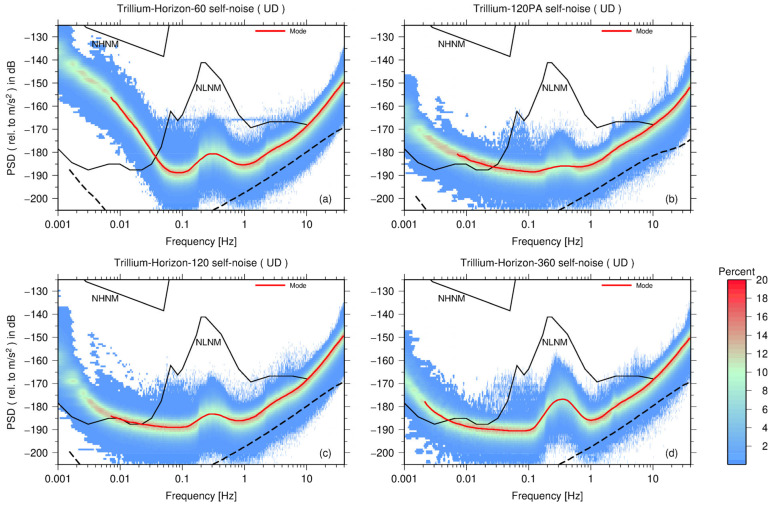
PDF distributions of self-noise PSD (on U–D channel) of four types of seismometers from Nanometrics Inc. (**a**) Trillium-Horizon-60, (**b**) Trillium-120PA, (**c**) Trillium-Horizon-120, and (**d**) Trillium-Horizon-360. The red curve is the mode curve of the PSD after 1/8 octave smoothing. The black dashed curve represents the EDAS-24GN self-noise.

Figure 4 displays the PDF presentations of self-noise PSD in vertical direction of four types of seismometers from Nanometrics Inc., Kanata, ON, Canada (Nanometrics). The Trillium-120PA is a portable broadband instrument, and the ground motion response has −3 dB corners at 120 s and 175 Hz. The Trillium-Horizon-60 is a broadband sensor with a low-end cutoff frequency of 60 s. The Trillium-Horizon-120 is also a broadband sensor with a flat velocity response from 120 s to 150 Hz. Finally, the Trillium-Horizon-360 is an ultra-broadband instrument with low-end and high-end cutoff frequency of 373 s and 155 Hz, respectively.

## 4. Discussion

**Figure 5:**
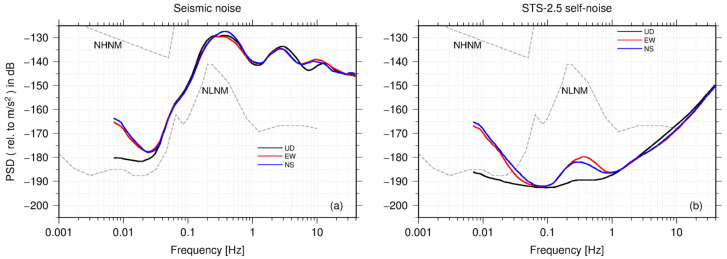
Comparison of background noise models by STS-2.5 and STS-2.5 self-noise models for U–D, E–W, and N–S channels. (**a**) Background noise models of three channels and (**b**) STS-2.5 self-noise models of three channels. The PSD curve is the mode of the PDF distribution.

We first compared the seismic ambient noise of the three channels measured by STS-2.5 with the self-noise of the three channels of STS-2.5. The curves of the noise PSDs are the mode of the PDF. As shown in Figure 5a, when the frequency is greater than 0.03 Hz, the seismic ambient noise levels of the three channels are roughly the same; however, when the frequency is lower than 0.03 Hz, a significant difference occurs between the horizontal and vertical ambient noise. For a long-period noise (>0.01 Hz), the instrumental self-noise could be the major source of noise [12,19]. At 0.007 Hz, the PSD of the horizontal ambient noise is approximately −165 dB and the vertical noise is -180 dB, slightly higher than NLNM. 

Figure 5b shows an instrumental self-noise model of the three STS-2.5 channels. When the frequency is higher than 1 Hz, the self-noise levels of the three channels are approximately the same; however, when the frequency is between 0.1 and 1 Hz, the self-noise levels of the vertical (U–D) and horizontal (E–W and N–S) ones vary significantly. The misalignment between the seismometers may lead to substantial variance in the estimation of the instrumental self-noise, and this variance is more pronounced when the noise data have a high SNR [13]. The seismic ambient noise has a predominant peak between 0.1 and 1 Hz, where marine microseisms dominate. Since the difference between the background noise and the sensor self-noise reaches its maximum close to the peak, such as 55 dB in the vertical direction, the error caused by the misalignment is also significant. At 0.3 Hz, the PSD of the E–W channel is 10 dB higher than that of the U–D channel. Compared with the vertical self-noise, the trend of the significantly higher horizontal self-noise is consistent with that of the ambient noise. Therefore, the difference between the horizontal and vertical self-noises in the microseism band (between 0.1 and 1 Hz) is ascribed mainly to the misalignment between the sensors in the horizontal direction. The reasons for misalignment of the same orientation between seismometers are mainly from two aspects: (1) the orientation deviation due to the alignment accuracy of the sensors, which is limited by the manufacturing process; (2) the alignment operation during the installation of the seismometers can also cause the orientation between the sensors to not be strictly in line. In real settings, when seismometers are installed, it is more difficult to maintain alignment in the horizontal than in the vertical direction, resulting in more significant alignment errors in the corresponding direction. In this study, we mainly examined the seismometer self-noise in the vertical direction. 

When the frequency is lower than 0.1 Hz, the PSD curve of vertical self-noise of STS-2.5 is always lower than NLNM. When the frequency is lower than 0.06 Hz, the PSD curve of horizontal self-noise increases rapidly along with the decreasing frequency. The self-noise curve of the E–W channel intersects the NLNM curve at 0.03 Hz and, at 0.007 Hz, the PSD value is close to the background noise of the E–W channel mentioned above. We also noticed that as the frequency decreased from 0.06 to 0.03 Hz, the self-noise of the seismometer in the horizontal direction presented an opposite trend to that of the seismic ambient noise. Therefore, in the low frequencies (<0.06 Hz), the self-noise in the horizontal direction that increases significantly may not be a calculation error due to misalignment in the horizontal direction. Given that the tunnels in the Malingshan Seismic station were not airtight, barometric pressure variations could substantially influence the self-noise of the long-period seismometers [12,20,21]. Relative to the vertical direction, the sensor horizontal direction is more likely to be affected by local changes in wind and pressure [12].

**Figure 6:**
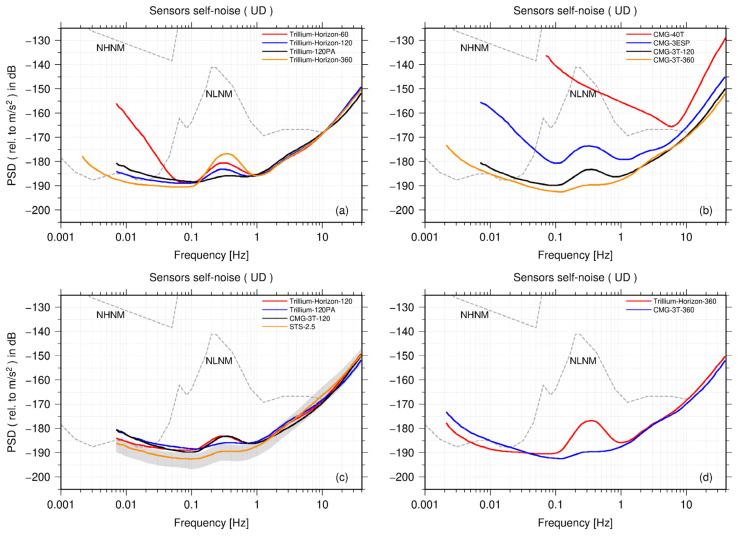
Comparison of self-noise models on U–D channels for nine different types of seismic instruments. (**a**) Four seismic instruments from Nanometrics Inc., (**b**) four seismic instruments from Güralp, (**c**) four seismometers with a low-end cutoff frequency of 120 s, and (**d**) two ultra-broadband seismometers. The self-noise PSD curve is the mode of the PDF distribution. The gray band in (**c**) is the 68% confidence interval of STS-2.5 self-noise PSDs.

Furthermore, we compared the vertical self-noise models of the nine types of seismometers. Figure 6a shows the self-noise model of four types of seismometers from Nanometrics Inc. When the frequency is higher than 1 Hz, the self-noise levels of the four seismometers are roughly the same. At low frequencies (<0.06 Hz) and frequencies between 0.1 and 1 Hz, the noise level of the Trillium-Horizon-60 is higher than that of the Trillium-Horizon-120. Regarding the Trillium-Horizon-120 and Trillium-120PA, the self-noise PSD curve presents a trade-off between the microseism band and the low-frequency band. The PSD curve of the Trillium-120PA is slightly higher than that of the Trillium-Horizon-120 between 0.13 and 1 Hz and is relatively lower below 0.13 Hz. This trade-off phenomenon is most pronounced for the ultra-broadband Trillium-Horizon-360. When the frequency is between 0.13 and 1 Hz, the self-noise level of the Trillium-Horizon-360 is even higher than that of the Trillium-Horizon-60. In particular, at 0.35 Hz, the PSD value of the Trillium-Horizon-360 is 9 dB higher than that of the Trillium-120PA. However, when the frequency is lower than 0.13 Hz, the Trillium-Horizon-360 has the lowest self-noise level of all the tested seismometers. Although the Trillium-Horizon-360 has a better performance at low frequencies (<0.13 Hz), the comparatively higher PSDs in the microseism band indicate a large alignment error in the vertical direction among the three instruments.

Figure 6b presents the self-noise model of four seismometers from Güralp. The self-noise level of the short-period seismometer CMG-40T is significantly higher than that of the other broadband seismometers. At a frequency between 0.1 Hz and 1 Hz, the PSD curve of the CMG-40T is close to that of the NLNM, which is consistent with that of Tasič and Runovc [7]. The short-period seismometer can be used for seismic wave arrival-time picking, earthquake location, and magnitude estimation. As regards analyzing longer-period seismic waveforms or research on low-noise seismic data (such as using teleseismic surface waves for imaging the deep structure of the Earth and tomography based on seismic ambient noise and microseismic monitoring), the self-noise of the sensor must be lower than the ambient noise of the Earth in the frequency band of interest and as low as possible below the NLNM. In such instance, a broadband seismometer with a lower noise level is required, as short-period sensors with higher noise levels are not suitable. Across the entire frequency range, the self-noise level of the broadband CMG-3ESP is significantly lower than that of CMG-40 but higher than that of the CMG-3T-120. At a frequency between 0.05 Hz and 9 Hz, the PSD curve of the CMG-3ESP is lower than the NLNM curve. Considering that the low-end cutoff frequencies of the CMG-3ESP and CMG-3T-120 are 60 s and 120 s, respectively, the difference between the self-noises of the two seismometers gradually amplifies along with the decreasing frequency. At a frequency higher than 2 Hz, the self-noise of the CMG-3T-360 is the same as that of the CMG-3T-120; however, when the frequency is lower than 2 Hz, the self-noise level of the CMG-3T-360 is lower than that of the CMG-3T-120. Within the microseism band, the self-noise PSD curve of the CMG-3T-360 is apparently not coherent with the seismic ambient noise; while, at 0.02 Hz, the self-noise PSD curve of the CMG-3T-360 intersects the NLNM, and when the frequency is between 0.01 Hz and 0.02 Hz, it is close to the NLNM. The frequency ranges in which the self-noise of different sensors is below the NLNM are given in Appendix C.

We also compared the self-noise levels of four very broadband seismometers of which the low-end cutoff frequency is 120 s, as shown in Figure 6c. When the frequency is higher than 2 Hz, the self-noise PSD curves of the four seismometers are in good agreement. At high frequencies, as shown in Figure 2, Figure 3 and Figure 4, the self-noise PSDs of the EDAS-24GN reach the lower limit of PDF distributions of seismometers, and thus the datalogger noise level may limit the estimation of the sensor self-noise in this case. Moreover, the gray area in Figure 6c is the 68% confidence interval for the self-noise PSD of STS-2.5, and the standard deviation around the mode curve of STS-2.5 is between 3 and 4 dB. When the frequency is less than 1 Hz, the PSD curves of the CMG-3T-120, Trillium-Horizon-120, and Trillium-120PA are higher than the confidence interval of the STS-2.5, and the self-noise level of the STS-2.5 is superior to that of the other sensors.

Finally, as shown in Figure 6d, we compared the self-noise levels of two ultra-broadband seismometers (CMG-3T-360 and Trillium-Horizon-360). When the frequency is higher than 1 Hz, the self-noise curves of two sensors are roughly the same; however, between 0.1 and 1Hz, the self-noise level of the Trillium-Horizon-360 is higher and is consistent with the trend of the NLNM. Therefore, the alignment error of the Trillium-Horizon-360 in the vertical direction could be greater than that of the CMG-3T-360. At low frequencies (<0.03 Hz), however, the self-noise level of the Trillium-Horizon-360 is superior to that of the CMG-3T-360. When the frequency is between 0.002 and 0.01 Hz, the PSD value of the Trillium-Horizon-360 is approximately 4 dB less than that of the CMG-3T-360.

## 5. Conclusions

We obtained the self-noise models of nine types of seismometers using PDF representation and based on the continuous waveform data recorded at the Malingshan Seismic Station from 25 November 2018–25 March 2019. For the STS-2.5, the significantly higher horizontal components of the self-noise in the vicinity of ocean microseisms coincide with the trend of the background noise, which could be ascribed to deviation in calculation caused by the misalignment between the sensors in the horizontal direction. At low frequencies, air pressure fluctuations could have substantial effects on the long-period self-noise of the horizontal components of the seismometer. At 0.007 Hz, the instrumental self-noise of the horizontal component is close to the seismic background noise and may be the dominant source of the noise recorded.

Regarding the Trillium-Horizon-120, Trillium-120PA, and Trillium-Horizon-360 seismometers from Nanometrics Inc., the self-noise PSD curves presented a trade-off between the microseism band and low-frequency band. When the level of self-noise in the microseism band is high, the self-noise at low frequencies is relatively low. As regards the seismometers from Güralp, the self-noise level of short-period CMG-40T is higher than that of the other broadband seismometers. When the frequency is between 0.1 and 1 Hz, the PSD curve of CMG-40T is close to the NLNM. Therefore, short-period seismometers are not suitable for research on low-noise seismic data. When the frequency is lower than 2 Hz, the self-noise levels of the ultra-broadband CMG-3T-360 outperform other seismometers from Güralp. 

When the frequency is lower than 1 Hz, the STS-2.5 self-noise level of the vertical component is 3 dB to 4 dB lower than that of the other three seismometers of which the low-end cutoff frequencies are 120 s. The difference in the self-noise of the two ultra-broadband seismometers characterizes the difference in the performances of different seismometers. The self-noise of the CMG-3T-360 is superior between 0.1 and 1 Hz, and the self-noise level of the Trillium-Horizon-360 is lower at low frequencies (<0.1 Hz). Except for the CMG-40T and CMG-3ESP, the self-noise levels of the other seven broadband seismometers at high frequencies (>2 Hz) are generally consistent. The noise of the EDAS-24GN could have constraints on the estimation of seismometer self-noise at high frequencies.

The self-noise models of nine different types of seismic instruments can provide a better understanding of geoscience research and help to avoid potential uncertainties when using the seismic data from these sensors for geophysical interpretation. However, the nine seismometers in this study cannot cover all types within China. The self-noise levels of more types of seismometers will be studied in the future.

## Figures and Tables

**Table 1:** Calculation parameters required for PSD of nine models of seismometers.

Category	Sensor Type	Low Cutoff Frequency (s)	Voltage Sensitivity (V/m/s)	Sampling Rate (sps)	Segment Length (h)	Segment Overlap (%)	Sub-Segment Length (s)	Sub-Segment Overlap (%)
Short-Period	CMG-40T-1	2 s	2000	100	1	50	163.84	87.5
Broadband	CMG-3ESP	60 s	2000	100	1	50	1310.72	87.5
	Trillium-Horizon-60	60 s	2000	100	1	50	1310.72	87.5
VeryBroadband	CMG-3T-120	120 s	2000	100	1	50	1310.72	87.5
	Trillium-Horizon-120	120 s	2000	100	1	50	1310.72	87.5
	Trillium-120PA	120 s	2000	100	1	50	1310.72	87.5
	STS-2.5	120 s	1500	100	1	50	1310.72	87.5
Ultra-Broadband	CMG-3T-360	360 s	2000	100	4	75	5242.88	87.5
	Trillium-Horizon-360	373 s	2000	100	4	75	5242.88	87.5

**Figure A1:**
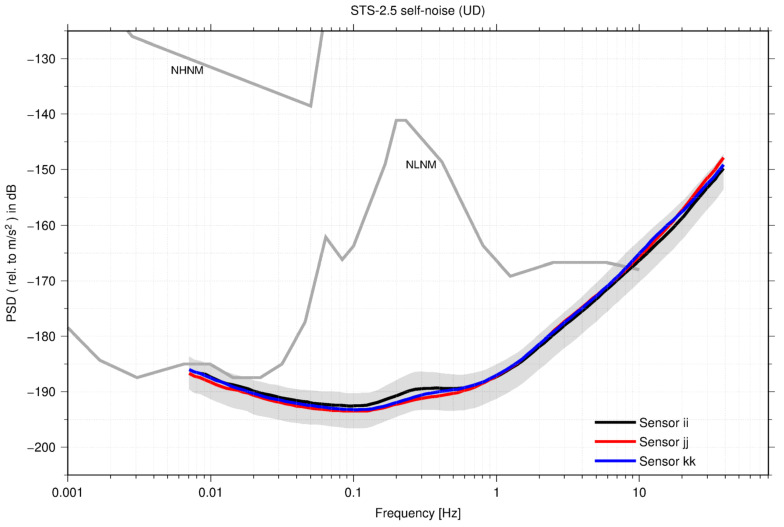
Self-noise models of three seismometers (*ii*, *jj*, and *kk*) of the STS-2.5 on U–D channels. The black, red, and blue curves are the modes of the PSD after 1/8 octave smoothing. The gray band is the 68% confidence interval of self-noise PSDs for sensor *ii*.

**Figure A2:**
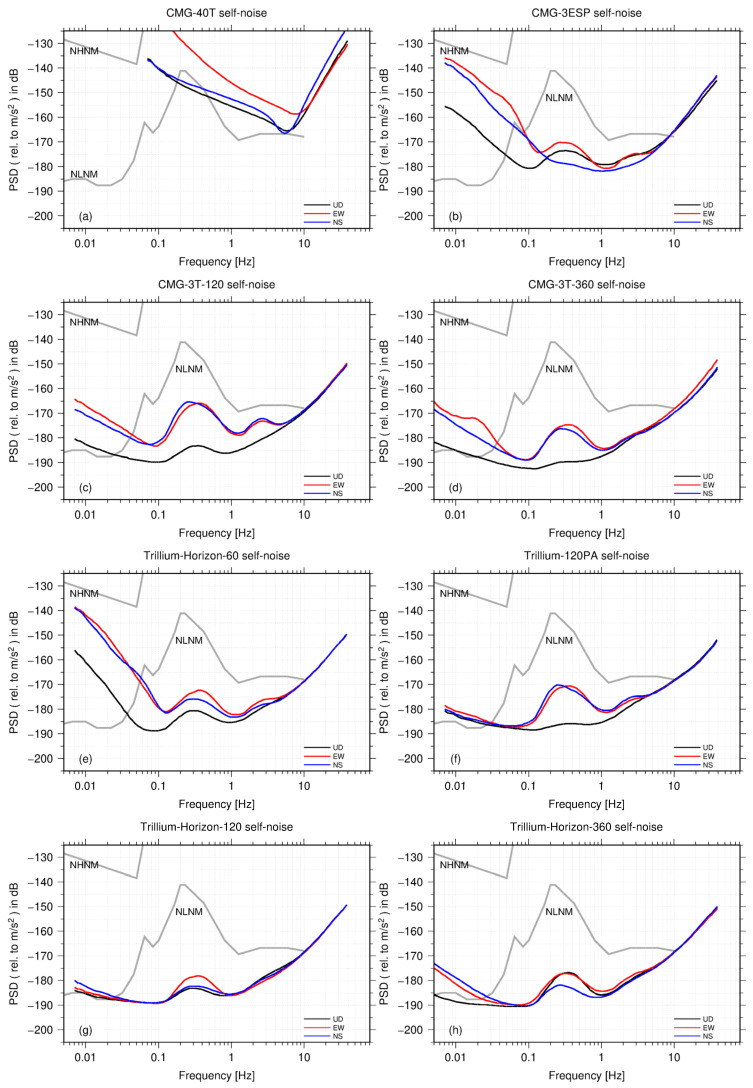
Self-noise models of three channels (U–D, E–W, and N–S) for different types of seismometers. The black, red, and blue curves are the modes of the PSD after 1/8 octave smoothing.

## Data Availability

All waveform data in this study were recorded at Malingshan Seismic Station in China and are available from us upon request.

## Appendix A

For each type of seismometer, we obtained self-noise models for each of the three sensors by the three-channel coherence analysis technique. Figure A1 is the self-noise models of the three sensors of the STS-2.5 on U–D channels. The self-noise levels of three seismometers are in good agreement, and we selected the self-noise model of sensor *ii* (black curve) in this paper.

## Appendix B

We obtained the self-noise models for three channels of different types of seismometers, as is shown in Figure A2. For most types of sensors, the self-noises on horizontal channels show similar features: (1) the higher noise levels on the horizontal channels (E–W and N–S) in the microseism band are caused by misalignment between the sensors; (2) the elevated noise on the horizontal channels at low frequencies (mostly <0.06 Hz) may originate from the local variation of atmospheric pressure.

## Appendix C

As is shown in Table A1, for each type of sensor, we give the values of low frequency and high frequency, which represent two intersections of the self-noise PSD curve and NLNM, and the self-noise level of seismometer is below NLNM, between the two frequencies. It is worth noting that the low-frequency value of the STS-2.5 is the lower limit in the studied frequency range, and the actual low-frequency intersection is less than 0.007 Hz.

**Table A1:** Frequency of the intersection of self-noise PSD curve and NLNM for different types of seismometers.

Category	Sensor Type	Low Frequency (Hz)	High Frequency (Hz)
Short-period	CMG-40T-1	0.180	0.5
Broadband	CMG-3ESP	0.050	9.0
	Trillium-Horizon-60	0.040	10.0
Very broadband	CMG-3T-120	0.025	10.0
	Trillium-Horizon-120	0.020	10.0
	Trillium-120PA	0.030	10.0
	STS-2.5	0.007	9.0
Ultra-broadband	CMG-3T-360	0.020	10.0
	Trillium-Horizon-360	0.005	10.0
